# NADPH oxidase–associated oxidative stress and ferroptosis-related injury in calcium oxalate-exposed renal tubular epithelial cells: pharmacological evidence for PI3K/AKT involvement

**DOI:** 10.1080/0886022X.2026.2750582

**Published:** 2026-10-30

**Authors:** Ming Xu, Ning Li, Zhenyu Geng

**Affiliations:** Department of Emergency, The Third People’s Hospital of Hefei, Hefei, China

**Keywords:** Calcium oxalate, ferroptosis, NADPH oxidase, reactive oxygen species, PI3K/AKT signaling, NRK-52E cells

## Abstract

**Background/Objectives:**

Calcium oxalate (CaOx) crystals contribute to renal tubular injury in nephrolithiasis, but the relationship among NADPH oxidase (NOX), oxidative stress, ferroptosis-related injury, and PI3K/AKT signaling remains incompletely defined. We examined CaOx-induced injury in NRK-52E renal tubular epithelial cells and tested the effects of pharmacological NOX and PI3K inhibition on oxidative and ferroptosis-related readouts.

**Methods:**

NRK-52E cells were exposed to CaOx (0–10 mM) and assessed by CCK-8, DCFH-DA flow cytometry, NOX activity assay, NADPH and Fe^2+^ assays, GSH and malondialdehyde (MDA) assays, quantitative real-time PCR (qRT-PCR), Western blotting, and transcriptome sequencing. Ferrostatin-1 (Fer-1, 2 μM), Erastin (10 μM), the NOX inhibitor GKT137831 (10 μM), and the PI3K inhibitor LY294002 (10 μM) were used as pharmacological probes.

**Results:**

CaOx reduced cell viability and increased intracellular ROS in a concentration-dependent manner. At 1 mM, CaOx was associated with lower NADPH and GSH, higher Fe^2+^, MDA, and ROS, increased NOX activity and NOX4 mRNA and protein expression, reduced GPX4, and increased ACSL4. Fer-1 ameliorated, whereas Erastin aggravated, several ferroptosis-associated readouts. CaOx also increased the p-PI3K/PI3K and p-AKT/AKT ratios. GKT137831 and LY294002 each attenuated CaOx-associated oxidative stress and ferroptosis-related phenotypes in this cell model.

**Conclusions:**

The findings support a contribution of NOX-associated oxidative stress and ferroptosis-related injury to CaOx-exposed NRK-52E cells. Pharmacological experiments indicate that NOX and PI3K/AKT signaling participate in this phenotype. These findings are limited to an *in vitro* cell model and require validation in animal and human-relevant systems.

## 1. Introduction

Nephrolithiasis affects about 7–13% of people in Western countries and 5–9% in Asia. Calcium oxalate (CaOx) is the main component of most kidney stones and is present in nearly 80% of cases [1]. Once CaOx crystals deposit on renal tubular epithelial cells, they disrupt intracellular redox homeostasis, provoke inflammatory signaling, and injure the epithelium. Damaged cells then provide a more favorable surface for crystal retention, which in turn promotes aggregation and calcification [2].

Reactive oxygen species (ROS) play a central role in this process. Clinical studies and experimental work both show that ROS levels rise in urine and renal tissue in nephrolithiasis, and greater ROS accumulation is accompanied by more severe tubular injury [3]. CaOx crystals also activate the NLRP3 inflammasome through ROS-dependent pathways, which further intensifies renal inflammation [4]. However, the contribution of NOX-derived oxidative stress to CaOx-induced tubular epithelial injury remains incompletely defined.

NADPH oxidase (NOX) is an enzymatic source of superoxide in renal tubular epithelial cells [5–7]. Prior work implicates NOX-derived oxidative stress in renal injury [8], but its contribution to CaOx-exposed tubular cells requires direct experimental testing. Because NOX-derived ROS can promote lipid peroxidation, ferroptosis is a plausible component of the downstream injury response. We therefore evaluated NOX activity, NOX4 mRNA and protein expression, and the effects of pharmacological NOX inhibition in the present cell model.

Ferroptosis is an iron-dependent form of regulated cell death driven by lipid peroxidation and loss of GPX4 activity, and it is mechanistically distinct from apoptosis and necrosis [9,10]. In the kidney, ferroptosis has been linked to tubular injury in acute kidney injury, cisplatin nephrotoxicity, and diabetic nephropathy [11–13]. Evidence also connects this pathway with CaOx-induced tubular damage. High oxalate levels activate ferroptosis-related pathways in renal tubular cells [14], and Ferrostatin-1 (Fer-1) lessens oxalate-induced cell death and crystal deposition *in vitro* [15]. Transcriptomic analyzes from patients with nephrolithiasis also show altered expression of ferroptosis-associated genes [16]. Autophagy has likewise been reported to facilitate ferroptosis-related responses after oxalate exposure [17]. However, the relationship between NOX-associated oxidative stress and ferroptosis-related changes after CaOx exposure remains incompletely defined.

PI3K/AKT signaling regulates cell survival, redox homeostasis, and ferroptosis-associated processes in a context-dependent manner [18–21]. Its functional direction in CaOx-associated tubular injury remains uncertain, making it important to distinguish pathway phosphorylation from functional pathway involvement.

In the present study, we used NRK-52E rat renal tubular epithelial cells to examine CaOx-induced injury, oxidative stress, ferroptosis-related readouts, and PI3K/AKT signaling. We combined pharmacological modulation with qRT-PCR, transcriptomic, and protein-level analyzes. Because this is an *in vitro* pharmacological study, the findings are interpreted as evidence of pathway involvement rather than proof of an isoform-specific or linear causal cascade.

## 2. Materials and methods

### 2.1. Reagents and instruments

#### 2.1.1. Reagents

Calcium oxalate (CaOx; Sigma-Aldrich/Merck KGaA, Darmstadt, Germany; catalog no. 455997-5 G); fetal bovine serum (FBS; Gibco, A3160802); DMEM culture medium (Gibco, C11965500BT); 0.25% trypsin (BIOMEDICAL, B2048); penicillin-streptomycin (Gibco, 15140122); PBS (Gibco, C10010500BT); CCK-8 (BIOMEDICAL, B1099); DCFH-DA ROS detection kit (Solarbio, CA1410); NADPH content assay kit (Solarbio, BC1100); cellular iron content assay kit (Solarbio, BC5315); NOX assay kit (Solarbio, BC0635); GSH assay kit (Elabscience, E-BC-K030-M); MDA assay kit (Elabscience, E-BC-K814-M); high-efficiency RIPA lysis buffer (Solarbio, R0010); BCA protein assay kit (Beyotime, P0010); SweScript RT II First Strand cDNA Synthesis Kit with gDNA Remover (Servicebio, G3333); ArtiCanA SYBR qPCR Mix (Servicebio, G3326); Ferrostatin-1 (Fer-1; working concentration, 2 μM), Erastin (working concentration, 10 μM), GKT137831 (GLPICO; working concentration, 10 μM), and LY294002 (working concentration, 10 μM). Primary antibodies included PI3K, p-PI3K p85α Tyr607, p-AKT Ser473, AKT, NOX4, GPX4, ACSL4, OPN, MCP-1, and GAPDH.

#### 2.1.2. Instruments

Biological safety cabinet (Thermo, 1300-A2); CO_2_ incubator (Thermo, 3111); inverted fluorescence microscope (Mshot, MF53); low-speed centrifuge (Xiangyi, L400); refrigerated high-speed centrifuge (Xiangyi, TGL-16K/TGL-20M); microplate reader (Tecan, Infinite F50); NanoPhotometer spectrophotometer (Implen); QuantReady K9600 real-time PCR system (Hangzhou Suizhen Biotechnology Co., Ltd.); mini vertical electrophoresis system (Hongjee, HT-Mini04); mini transfer electrophoresis system (Hongjee, HT-ZY02); chemiluminescence imaging system (Hangzhou Shenhua, SH-523); constant-temperature metal bath (DLAB, H120-S); constant-temperature magnetic stirrer (Guoyu, 79HW-1); rocker shaker (SCILOGEX, SLK-O3000-S); dual-stable electrophoresis power supply (Liuyi, DYY-6C); analytical balance (Ohaus, PX224ZH/E).

### 2.2. Cell culture and passaging

NRK-52E rat renal tubular epithelial cells were maintained in DMEM supplemented with 10% FBS and 1% penicillin-streptomycin at 37 °C in a humidified 5% CO_2_ atmosphere. Cells were passaged upon reaching 80%–90% confluency. Briefly, culture medium was aspirated, cells were washed once with PBS, and 0.25% trypsin was added (1 mL per 60-mm dish; 2 mL per 100-mm dish). After a 2-min incubation at 37 °C, when approximately 70%–80% of cells had detached, trypsinization was terminated by adding twice the volume of complete culture medium. The cell suspension was centrifuged at 1000 rpm for 3 min, the supernatant was discarded, and the cells were resuspended and subcultured at a 1:3 ratio.

### 2.3. CCK-8 cell viability assay

Cells were seeded in 96-well plates, pre-cultured for 24 h, and then treated as indicated. CCK-8 reagent (10 μL/well) was added, and the cells were incubated for an additional 2 h at 37 °C before absorbance was measured at 450 nm. For the CaOx concentration-response experiment, cells were exposed to 0–10 mM CaOx. For time-course experiments, separate wells were prepared for each measurement time point, and the same wells were not repeatedly measured over time. Where normalized time-course data were presented, OD_450_ values were normalized to the corresponding day-1 value within each treatment group.

### 2.4. Intracellular ROS detection

Intracellular ROS were quantified using the DCFH-DA fluorescent probe. Following drug treatment, DCFH-DA was diluted to 10 μmol/L in serum-free DMEM and applied to cells for 20 min at 37 °C with gentle mixing every 3–5 min. Cells were washed three times with serum-free medium to remove extracellular probe, collected, and analyzed by flow cytometry (excitation: 488 nm; emission: 525 nm; FITC channel).

### 2.5. NADPH content assay

Intracellular NADPH was quantified using the Solarbio BC1100 kit. Five million cells per sample were resuspended in 0.5 mL acidic extraction buffer, subjected to sonication (200 W; 2-s pulse/1-s interval; 1 min total), boiled for 5 min, ice-cooled, and centrifuged at 10,000 × g, 4 °C for 10 min. A 200-μL aliquot of supernatant was neutralized with an equal volume of alkaline extraction buffer, re-centrifuged, and the final supernatant was assayed at 570 nm according to the kit instructions. NADPH content was calculated as: NADPH (nmol/10^4^ cells) = 0.002 × x_2_, where x_2_ is the sample concentration (nmol/mL) derived from the standard curve. Intracellular NADPH content was treated as a redox/metabolic readout and was not used as a surrogate measure of NOX activity.

### 2.6. Intracellular iron content assay

Intracellular Fe^2+^ was quantified using the Solarbio BC5315 kit based on the chromogenic reaction of Fe^2+^ with o-phenanthroline under mildly acidic conditions. A total of 5 × 10^6^ cells were mixed with 0.5 mL extraction buffer and sonicated in an ice-water bath (200 W; 3 s on/7 s off; 30 cycles). The lysate was centrifuged at 8,000 × g for 10 min at 4 °C, and the supernatant absorbance was measured at 510 nm. Iron content (ng/10^4^ cells) was calculated according to the kit formula: 27.922 × (ΔA_sample/ΔA_standard).

### 2.7. Western blot

Cells were lysed on ice for 30 min in RIPA buffer supplemented with PMSF (1:100), protease inhibitor cocktail (1:100), and phosphatase inhibitor cocktail (1:50). Lysates were clarified at 12,000 × g, 4 °C for 15 min, and protein concentrations were determined by BCA assay. Equal protein amounts were resolved by SDS-PAGE and transferred to methanol-activated PVDF membranes. After blocking, membranes were incubated with primary antibodies against NOX4, p-PI3K, PI3K, p-AKT, AKT, GPX4, ACSL4, OPN, MCP-1, and GAPDH, followed by HRP-conjugated secondary antibodies. Signals were visualized by ECL. For phosphorylation analyzes, p-PI3K and p-AKT were normalized to their corresponding total proteins. Other target proteins were normalized to GAPDH. Densitometric values obtained using different antibodies were not compared directly across proteins. Values for non-phosphorylated targets were expressed as target/GAPDH ratios and were not further rescaled by assigning the Blank group a value of 1.0.

### 2.8. Quantitative real-time PCR

Total RNA was extracted from NRK-52E cells using TRIzol reagent. After phase separation with chloroform, RNA was precipitated with isopropanol, washed twice with 75% ethanol, dissolved in DEPC-treated water, and stored at −80 °C until use. RNA concentration and purity were assessed using a NanoPhotometer spectrophotometer. Genomic DNA was removed, and first-strand cDNA was synthesized using the SweScript RT II First Strand cDNA Synthesis Kit with gDNA Remover (Servicebio, G3333) according to the manufacturer’s instructions. Reverse transcription was performed at 25 °C for 5 min, 42 °C for 30 min, and 85 °C for 5 s.

Quantitative real-time PCR was performed using ArtiCanA SYBR qPCR Mix (Servicebio, G3326) on a QuantReady K9600 real-time PCR system (Hangzhou Suizhen Biotechnology Co., Ltd.). Each 10-μL reaction contained 5.0 μL SYBR qPCR Mix, 0.2 μL forward primer (10 μM), 0.2 μL reverse primer (10 μM), 1.0 μL cDNA template, and 3.6 μL ddH_2_O. The cycling conditions were 95 °C for 60 s, followed by 40 cycles of 95 °C for 10 s and 60 °C for 20 s. Each cDNA sample was analyzed in technical triplicate. Relative NOX4 mRNA expression was normalized to GAPDH and calculated using the 2^−ΔΔCt method. Primer sequences were: NOX4 forward, 5′-GTGAACGCCCTGAACTTCTC-3′; NOX4 reverse, 5′-ATACCACCACCATGCAGACA-3′; GAPDH forward, 5′-TCCCATCACCATCTTCCAGG-3′; and GAPDH reverse, 5′-TGATGACCCTTTTGGCTCC-3′. Three independent biological replicate experiments were performed.

### 2.9. mRNA transcriptome sequencing

Total RNA was extracted from Blank and CaOx groups using TRIzol reagent. Samples passing quality control (RIN ≥ 7.0; OD_260_/_280_ = 1.8–2.1) were submitted for strand-specific library construction and Illumina paired-end sequencing (PE150). After quality filtering of raw reads and alignment to the rat reference genome, differential expression analysis was performed with DESeq2, using thresholds of |log_2_FC| > 1 and adjusted *p* < 0.05. DEGs were subjected to GO functional annotation and enrichment analysis and KEGG pathway analysis. Results were visualized as DEG statistics bar charts, volcano plots, hierarchical clustering heatmaps, GO classification charts, GO enrichment bubble plots, and KEGG pathway classification maps.

### 2.10. NOX, GSH, and MDA assays

For the NOX assay, cell lysates were prepared from approximately 5 × 10^6^ cells, clarified by centrifugation, and analyzed using the Solarbio BC0635 kit. NOX activity was expressed as U/mg protein according to the kit formula. GSH was measured with the Elabscience E-BC-K030-M kit at 405 nm, and MDA was measured with the Elabscience E-BC-K814-M kit at 532 nm, following the manufacturers’ instructions. Protein concentration was determined for normalization where applicable.

### 2.11. Pharmacological treatment groups

For Fer-1/Erastin experiments, cells were assigned to Blank, CaOx, CaOx + Fer-1 (2 μM), and CaOx + Erastin (10 μM) groups. For GKT137831 experiments, cells were assigned to Blank, CaOx, GKT137831, and CaOx + GKT137831 groups. Cells in the GKT137831-treated groups were pretreated with GKT137831 (10 μM) for 1 h and subsequently co-exposed to GKT137831 (10 μM) and CaOx (1 mM) for 24 h before ROS, NOX activity, GSH, MDA, and Western blot analyzes. For LY294002 experiments, cells were assigned to Blank, CaOx, LY294002, and CaOx + LY294002 groups. Cells in the LY294002-treated groups were pretreated with LY294002 (10 μM) for 1 h and subsequently co-exposed to LY294002 (10 μM) and CaOx (1 mM) for 24 h before ROS, GSH, MDA, and Western blot analyzes. For the CCK-8 time-course experiments, the same treatment concentrations were used and separate wells were designated for each time point.

For assays reported as *n* = 3, three independent biological replicate experiments were performed using separately cultured and treated cell preparations. Technical replicate wells, when used within an experiment, were not treated as independent biological replicates.

### 2.12. Statistical analysis

Statistical analyzes were performed using GraphPad Prism 9.0. Data are presented as means ± SD. Two-group comparisons were performed using unpaired Student’s t-tests. Multi-group comparisons at a single time point were analyzed by one-way ANOVA followed by Tukey’s *post hoc* test. For time-course cell-viability experiments, separate wells were used at each time point; therefore, the data were analyzed by two-way ANOVA with treatment and time as the two factors, followed by Tukey’s multiple-comparisons test. *p* < 0.05 was considered statistically significant. Statistical significance is indicated as *p < 0.05, **p < 0.01, ***p < 0.001, and ****p < 0.0001. Unless otherwise specified, n denotes independent biological replicate experiments.

## 3. Results

### 3.1. CaOx reduces NRK-52E viability, Increases ROS, and elevates NOX-related readouts

To define the injurious concentration range of CaOx in renal tubular cells, NRK-52E cells were exposed to 0, 0.1, 0.5, 1, 5, or 10 mM CaOx. Cell viability decreased while intracellular ROS increased with increasing CaOx concentration (Figure 1(A–D)), establishing a concentration-dependent injury profile. Based on these results, 1 mM CaOx was selected for subsequent experiments. At 1 mM CaOx, NOX activity was increased, accompanied by elevated NOX4 mRNA and protein expression (Figure 1(E–H)). In parallel, ACSL4 expression increased and GPX4 expression decreased (Figure 1(I,J)). These findings suggest an association between NOX-related oxidative stress and ferroptosis-related molecular changes in this model and provided the basis for subsequent pharmacological experiments.

**Figure 1.**
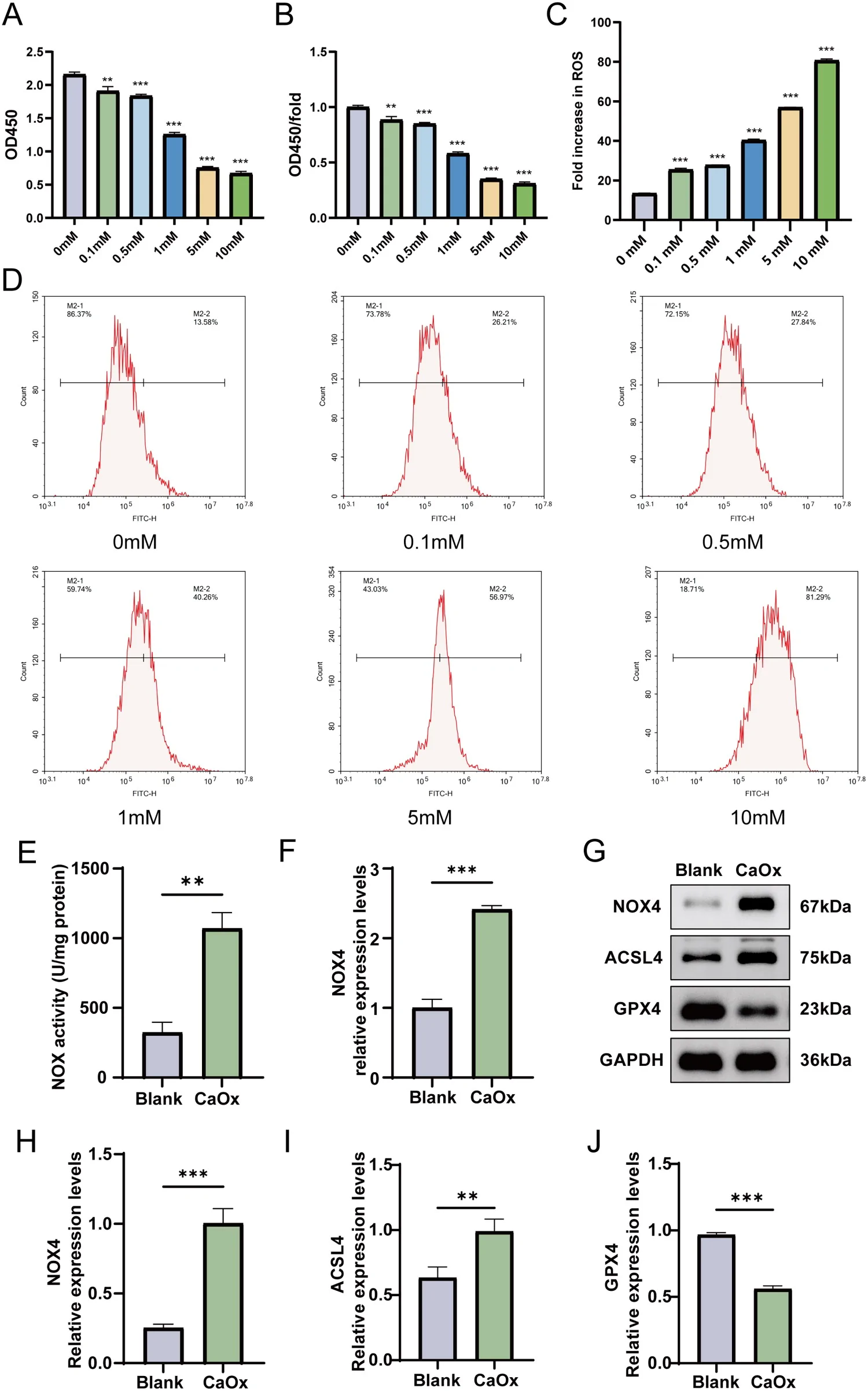
CaOx reduces NRK-52E cell viability, increases intracellular ROS, and elevates NOX-related readouts. (A) CCK-8 absorbance (OD_450_) following exposure to increasing concentrations of CaOx. (B) Relative CCK-8 viability normalized to the untreated group. (C, D) Quantification and representative DCFH-DA flow-cytometric profiles of intracellular ROS following CaOx exposure; the histograms are displayed in the same left-to-right order as the corresponding bars in (C). (E) NOX activity. (F) Relative NOX4 mRNA expression. (G) Representative Western blots of NOX4, ACSL4, and GPX4. (H–J) Densitometric quantification of NOX4, ACSL4, and GPX4 protein expression, respectively. Data are presented as means ± SD (*n* = 3 independent biological replicate experiments). **p < 0.01 and ***p < 0.001 versus the 0 mM control (A–C) or versus Blank (E, F, H–J); dose-response multi-group comparisons were analyzed by one-way ANOVA followed by Tukey’s *post hoc* test, whereas two-group comparisons were analyzed using unpaired Student’s t-tests.

### 3.2. Fer-1 and Erastin pharmacologically modulate ferroptosis-associated readouts in CaOx-exposed cells

To examine whether ferroptosis-related processes contribute to CaOx-induced injury, we evaluated the effects of pharmacological inhibition and induction of ferroptosis. NRK-52E cells were assigned to Blank, CaOx, CaOx + Fer-1 (2 μM), and CaOx + Erastin (10 μM) groups. Relative to Blank cells, CaOx alone reduced NADPH content, increased Fe^2+^ and ROS, and decreased viability (Figure 2(A–E)). Fer-1 counteracted, whereas Erastin aggravated, several of these readouts, producing opposite pharmacological effects on the CaOx-associated phenotype. Together with the GPX4/ACSL4 changes, these findings support a ferroptosis-associated component of CaOx-induced injury. However, this bidirectional pharmacological modulation does not exclude concurrent apoptosis, necroptosis, or other regulated cell-death programs.

**Figure 2.**
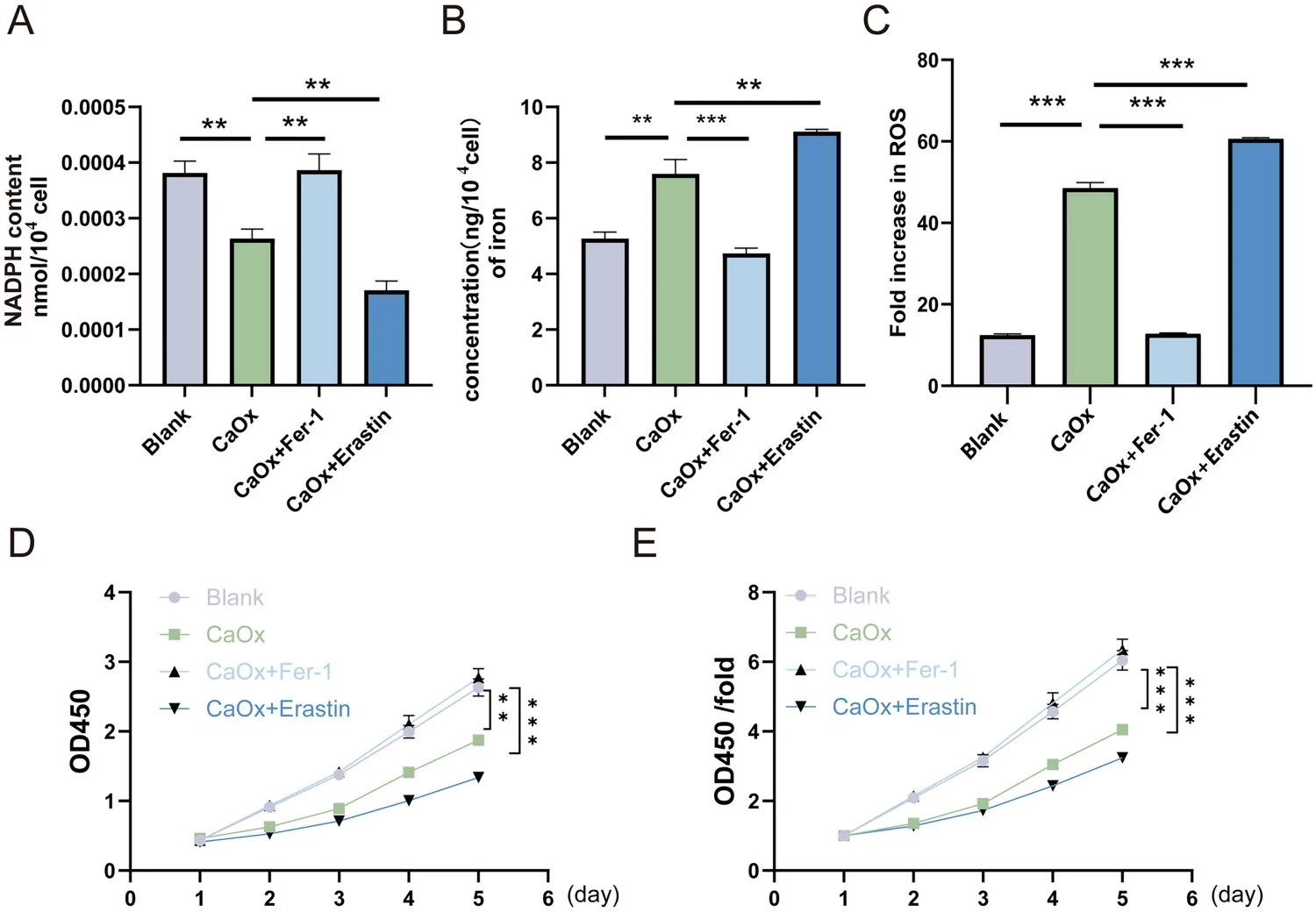
Fer-1 (2 μM) and Erastin (10 μM) pharmacologically modulate ferroptosis-associated readouts in CaOx-exposed NRK-52E cells. (A) Intracellular NADPH content. (B) Fe^2+^ content. (C) Intracellular ROS. (D) Time-course CCK-8 absorbance (OD_450_). (E) CCK-8 values normalized to the corresponding day-1 value within each treatment group. Data are presented as means ± SD (*n* = 3 independent biological replicate experiments). Panels A–C were analyzed by one-way ANOVA followed by Tukey’s *post hoc* test. Time-course data in panels D and E were analyzed by two-way ANOVA with treatment and time as factors, followed by Tukey’s multiple-comparisons test. *p < 0.05, **p < 0.01, and ***p < 0.001 for the comparisons indicated by the brackets (Blank versus CaOx, CaOx versus CaOx + Fer-1, and Blank versus CaOx + Erastin).

### 3.3. CaOx alters ferroptosis-related and inflammation-associated proteins

The biochemical changes were corroborated at the protein level. Western blot analysis showed that CaOx decreased GPX4 and increased ACSL4, while Fer-1 partially reversed and Erastin reinforced these changes (Figure 3). This pattern was consistent with the pharmacological effects observed in the viability and redox assays and was compatible with ferroptosis-related injury, although these markers are not, by themselves, specific for ferroptotic cell death.

**Figure 3.**
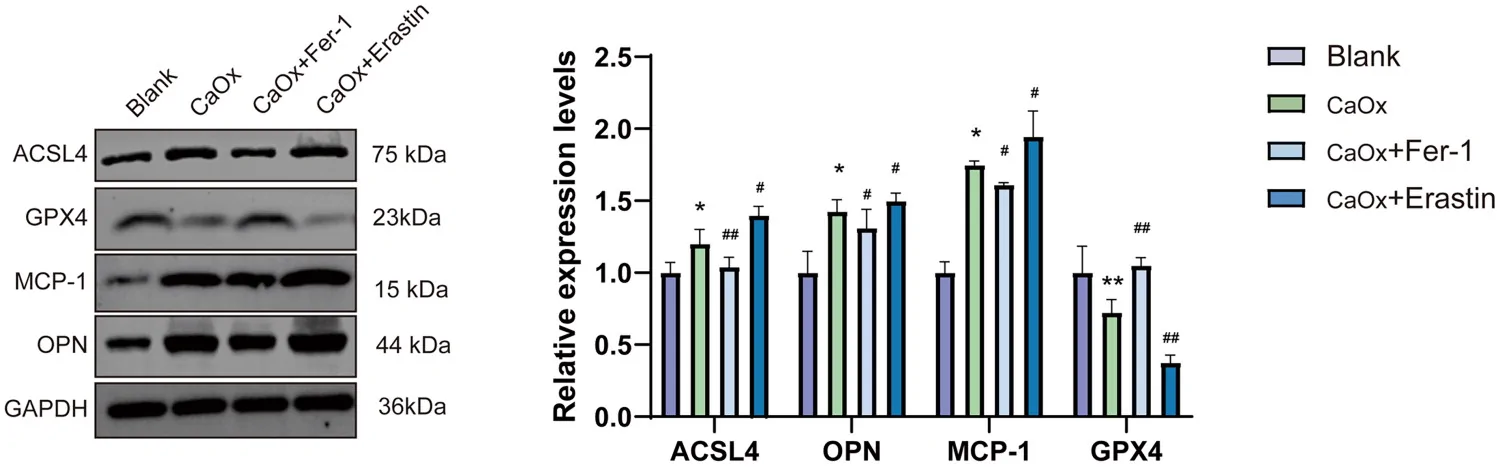
CaOx alters ferroptosis-related and inflammation-associated proteins. Representative Western blots and densitometric quantification of ACSL4, OPN, MCP-1, and GPX4 in Blank, CaOx, CaOx + Fer-1 (2 μM), and CaOx + Erastin (10 μM) groups. GAPDH served as the loading control. Densitometric values were expressed as target/GAPDH ratios and were not rescaled by assigning the Blank group a value of 1.0. Data are presented as means ± SD (*n* = 3 independent biological replicate experiments) and were analyzed by one-way ANOVA followed by Tukey’s *post hoc* test; *p < 0.05 and **p < 0.01 versus Blank, #*p* < 0.05 and ##*p* < 0.01 versus CaOx.

Ferroptosis-related injury has been associated with inflammatory signaling. Consistent with this association, OPN and MCP-1 were increased after CaOx exposure, and these changes were attenuated by Fer-1 and reinforced by Erastin (Figure 3). Inflammatory mediator expression thus covaried with ferroptosis-associated readouts, indicating an association between the two response patterns; however, these data do not establish ferroptosis as the upstream cause of the inflammatory response.

### 3.4. Transcriptome profiling identifies redox-, ferroptosis-, and PI3K/AKT-associated pathways

To extend these targeted readouts to an unbiased view of the CaOx-associated transcriptional landscape, we profiled NRK-52E cells after CaOx exposure (1 mM, 24 h) against untreated controls. Using |log_2_FC| > 1 and FDR < 0.05, we identified 153 differentially expressed genes (135 upregulated and 18 downregulated). The two conditions separated clearly in the clustering analysis, while the three replicate samples within each condition clustered together (Figure 4(A–C)), supporting the analytical consistency of the dataset at the single examined time point.

**Figure 4.**
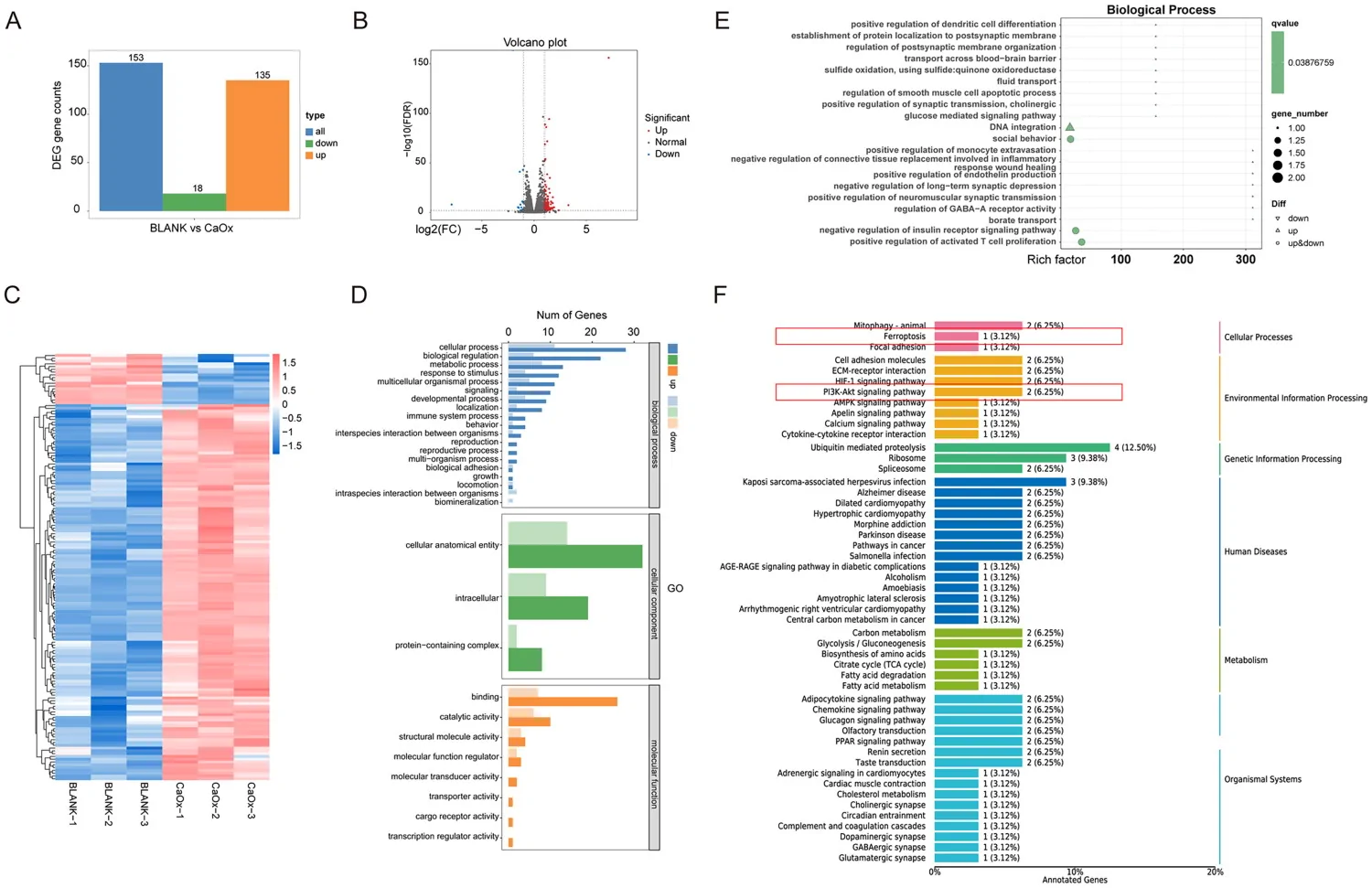
Transcriptome profiling of CaOx-treated and Blank NRK-52E cells. Differentially expressed genes were defined using |log_2_FC| > 1 and adjusted *p* < 0.05. (A) Numbers of up- and downregulated genes. (B) Volcano plot. (C) Hierarchical clustering. (D) GO classification. (E) GO enrichment bubble plot. (F) KEGG pathway classification. Ferroptosis and PI3K–AKT signaling are highlighted in panel F. Sequencing was performed with *n* = 3 independent biological replicates per group. The pathway-level transcriptomic findings were interpreted as hypothesis-generating and were further examined using protein-level and pharmacological approaches.

Functional analysis indicated that the differentially expressed genes were associated with multiple redox-related biological processes. GO analysis linked them to biological regulation, metabolic processes, response to stimuli, binding, and catalytic activity (Figure 4(D)), while oxidation–reduction-related processes were highlighted in the GO enrichment bubble plot (Figure 4(E)).

At the pathway level, KEGG analysis showed that the differentially expressed genes were mapped to multiple pathway categories, including ubiquitin-mediated proteolysis, ribosome-related pathways, ferroptosis, mitophagy, HIF-1 signaling, and PI3K–AKT signaling (Figure 4(F)). The presence of ferroptosis- and PI3K/AKT-associated pathway categories was consistent with the molecular phenotypes examined in subsequent experiments and provided a rationale for further protein-level and pharmacological analyzes. These transcriptomic observations are hypothesis-generating and do not independently establish functional pathway activation or causal relationships among the identified pathways.

### 3.5. CaOx increases PI3K and AKT phosphorylation

The presence of PI3K/AKT-associated signals in the transcriptomic analysis prompted further examination at the protein level. Western blotting showed higher p-PI3K (Tyr607) and p-AKT (Ser473) in CaOx-exposed cells, with the corresponding p-PI3K/PI3K and p-AKT/AKT ratios increased relative to Blank cells (Figure 5). CaOx exposure was therefore associated with increased PI3K/AKT phosphorylation in parallel with NOX upregulation and ferroptosis-associated changes. Because phosphorylation alone cannot define the functional direction of this signaling response, its relevance was further examined pharmacologically using the PI3K inhibitor LY294002.

**Figure 5.**
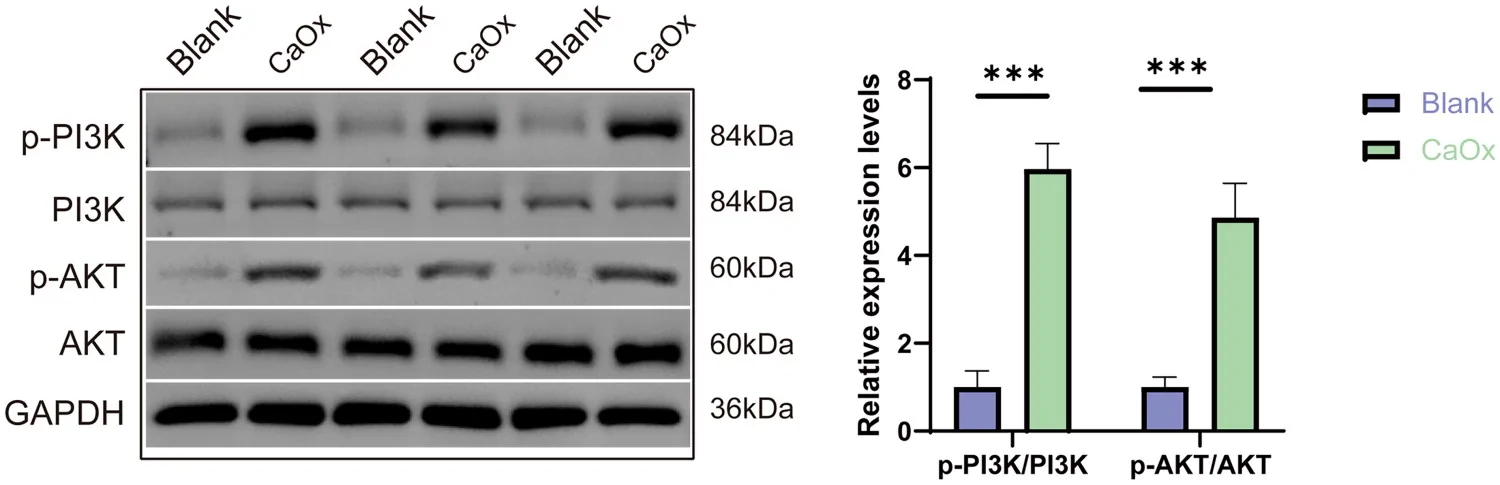
CaOx increases PI3K and AKT phosphorylation. Representative Western blots and p-PI3K/PI3K and p-AKT/AKT ratios in Blank and CaOx groups. GAPDH served as the loading control for the displayed blots. Phosphorylated proteins were quantified relative to their corresponding total proteins; band intensities from different target proteins were not compared directly. Data are presented as means ± SD (*n* = 3 independent biological replicate experiments) and were analyzed using unpaired Student’s t-tests; ***p < 0.001 versus Blank, as shown.

### 3.6. Pharmacological NOX inhibition attenuates CaOx-associated oxidative and ferroptosis-related changes

To further examine the involvement of NOX in CaOx-induced tubular epithelial cell injury, NRK-52E cells were assigned to Blank, CaOx, GKT137831, and CaOx + GKT137831 groups. Cells in the GKT137831-treated groups were pretreated with GKT137831 (10 μM) for 1 h and subsequently co-exposed to GKT137831 (10 μM) and CaOx (1 mM) for 24 h for the biochemical and molecular analyzes. GKT137831 co-treatment ameliorated the CaOx-induced reduction in cell viability over the observation period (Figure 6(A)). Intracellular ROS accumulation induced by CaOx was attenuated by GKT137831, as demonstrated by representative DCFH-DA flow-cytometric profiles and quantitative analysis (Figure 6(B,C)). In parallel, the CaOx-induced increase in NOX activity was significantly reduced following GKT137831 treatment (Figure 6(D)).

**Figure 6.**
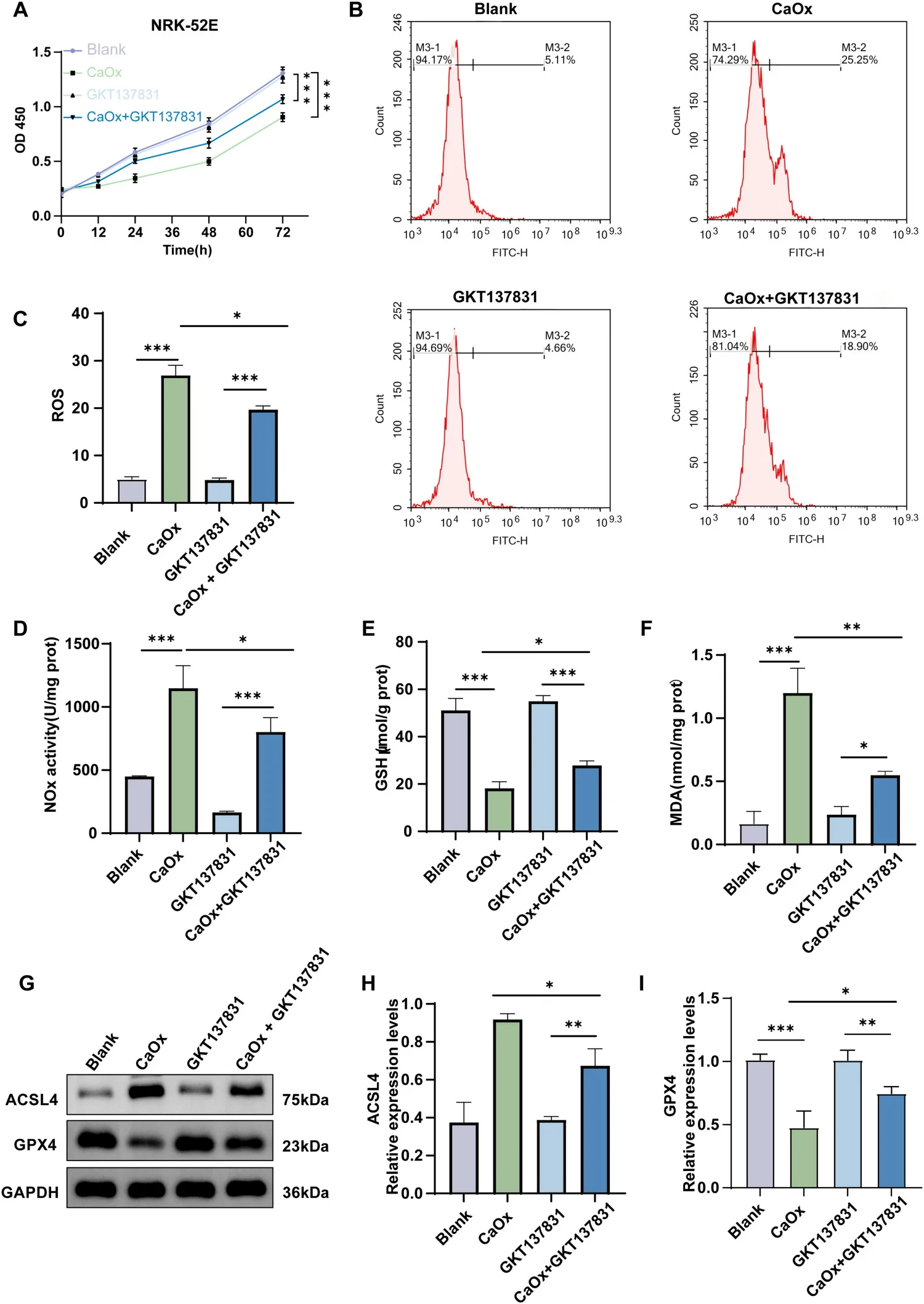
Pharmacological NOX inhibition with GKT137831 (10 μM) attenuates CaOx-associated oxidative and ferroptosis-related changes. (A) Time-course analysis of cell viability by CCK-8 assay. (B, C) Representative DCFH-DA flow-cytometry plots and quantitative analysis of intracellular ROS. (D) NOX activity. (E) GSH. (F) MDA. (G–I) Representative Western blots and densitometric quantification of ACSL4 and GPX4. Groups: Blank, CaOx, GKT137831 (10 μM), and CaOx + GKT137831 (10 μM). Data are presented as means ± SD (*n* = 3 independent biological replicate experiments). Time-course data in panel A were analyzed by two-way ANOVA with treatment and time as factors, followed by Tukey’s multiple-comparisons test. Single-time-point multi-group comparisons in panels C–I were analyzed by one-way ANOVA followed by Tukey’s *post hoc* test. *p < 0.05, **p < 0.01, and ***p < 0.001 for the comparisons indicated by the brackets.

Because oxidative stress and lipid peroxidation are closely associated with ferroptosis-related injury, intracellular antioxidant and lipid-peroxidation indices were further assessed. GKT137831 restored the CaOx-induced reduction in GSH levels (Figure 6(E)) and attenuated the increase in MDA content (Figure 6(F)). Western blot analysis further showed that GKT137831 partially reversed the CaOx-induced increase in ACSL4 expression and decrease in GPX4 expression (Figure 6(G–I)). Collectively, these findings indicate that pharmacological inhibition of NOX attenuates CaOx-associated oxidative stress, lipid peroxidation, and ferroptosis-related molecular changes in NRK-52E cells. However, because no isoform-specific genetic intervention was performed, these results do not establish NOX4 as the exclusive NOX isoform responsible for the observed effects.

### 3.7. PI3K inhibition attenuates CaOx-associated oxidative and ferroptosis-related changes

To investigate the functional involvement of PI3K/AKT signaling in CaOx-induced injury, NRK-52E cells were assigned to Blank, CaOx, LY294002, and CaOx + LY294002 groups. Cells in the LY294002-treated groups were pretreated with LY294002 (10 μM) for 1 h and subsequently co-exposed to LY294002 (10 μM) and CaOx (1 mM) for 24 h for the biochemical and molecular analyzes. LY294002 co-treatment ameliorated the CaOx-induced reduction in cell viability during the experimental period (Figure 7(A)). DCFH-DA flow cytometry further showed that PI3K inhibition attenuated CaOx-induced intracellular ROS accumulation (Figure 7(B,C)).

**Figure 7.**
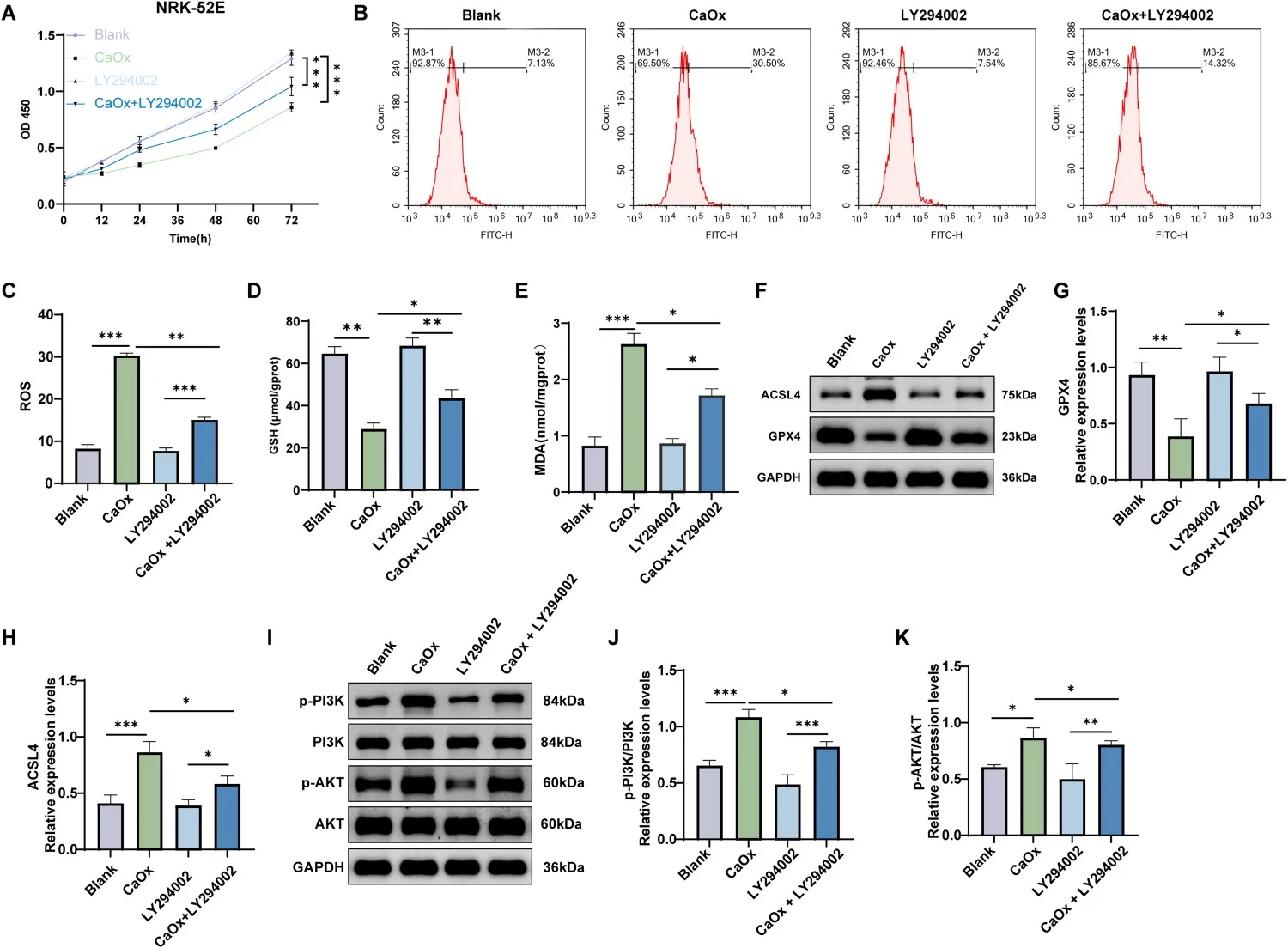
PI3K inhibition with LY294002 (10 μM) attenuates CaOx-associated oxidative and ferroptosis-related changes. (A) Time-course analysis of cell viability by CCK-8 assay. (B, C) Representative DCFH-DA flow-cytometry plots and quantitative analysis of intracellular ROS. (D) GSH. (E) MDA. (F–H) Representative Western blots and densitometric quantification of ACSL4 and GPX4. (I–K) Representative PI3K/AKT pathway blots and quantification of the p-PI3K/PI3K and p-AKT/AKT ratios. Groups: Blank, CaOx, LY294002 (10 μM), and CaOx + LY294002 (10 μM). Data are presented as means ± SD (*n* = 3 independent biological replicate experiments). Time-course data in panel A were analyzed by two-way ANOVA with treatment and time as factors, followed by Tukey’s multiple-comparisons test. Single-time-point multi-group comparisons in panels C–K were analyzed by one-way ANOVA followed by Tukey’s *post hoc* test. *p < 0.05, **p < 0.01, and ***p < 0.001 for the comparisons indicated by the brackets.

Consistent with the reduction in oxidative stress, LY294002 partially restored intracellular GSH levels (Figure 7(D)) and decreased MDA accumulation (Figure 7(E)) in CaOx-exposed cells. Western blot analysis demonstrated that LY294002 also attenuated the CaOx-associated increase in ACSL4 expression and partially restored GPX4 expression (Figure 7(F–H)). To confirm pharmacological inhibition of PI3K/AKT signaling, phosphorylation of PI3K and AKT was further examined. LY294002 reduced both the p-PI3K/PI3K and p-AKT/AKT ratios in CaOx-treated cells (Figure 7(I–K)).

Taken together, these pharmacological findings indicate that inhibition of PI3K/AKT signaling attenuates CaOx-associated oxidative stress, lipid peroxidation, and ferroptosis-related changes in NRK-52E cells. Under the present experimental conditions, the protective effects of LY294002 support pharmacological involvement of PI3K/AKT signaling in the CaOx-associated injury phenotype and favor an injury-associated functional direction. Nevertheless, these data do not establish the hierarchical relationship between PI3K/AKT and NOX signaling or determine whether PI3K/AKT directly regulates ferroptosis-related processes rather than broader cellular stress responses.

## 4. Discussion

This study combines molecular, biochemical, transcriptomic, and pharmacological data to characterize CaOx-associated injury in NRK-52E cells. CaOx exposure was accompanied by increased ROS, NOX activity, NOX4 mRNA and protein expression, lipid-peroxidation-associated MDA, and changes in GPX4 and ACSL4. These findings support an association between NOX-related redox disturbance and ferroptosis-related injury, while the intervention data support pharmacological pathway involvement rather than a complete causal cascade.

CaOx exposure was associated with Fe^2+^ accumulation, NADPH depletion, ROS elevation, and lower viability, and these readouts were modulated in opposite directions by Fer-1 and Erastin. Together with reduced GPX4 and increased ACSL4, the data support a ferroptosis-associated component of injury [9,22,23]. Importantly, intracellular NADPH depletion was interpreted only as a redox/metabolic change; direct evidence of NOX involvement was derived from the NOX activity assay, NOX4 expression, and pharmacological inhibition with GKT137831. Nevertheless, ROS elevation and impaired viability can accompany multiple regulated cell-death programs. We therefore do not infer from the Fer-1/Erastin experiments that ferroptosis is the exclusive or dominant mode of CaOx-induced cell death. Direct comparison with apoptosis and necroptosis interventions and readouts is required.

At the protein level, the CaOx-associated reduction in GPX4 and increase in ACSL4 are compatible with impaired detoxification of phospholipid hydroperoxides and increased susceptibility of membrane phospholipids to peroxidation [24–28]. The increased MDA and reduced GSH observed in the GKT137831 and LY294002 experiments provide additional biochemical evidence of lipid peroxidation and antioxidant-buffer disruption. However, we did not establish the temporal or causal ordering of NOX and PI3K/AKT signaling, nor did we directly measure lipid hydroperoxide species; mechanistic links beyond the measured endpoints remain to be tested.

CaOx increased OPN and MCP-1 expression, and these changes were modulated by Fer-1 and Erastin. This covariation is consistent with an association among oxidative injury, ferroptosis-related changes, and inflammatory signaling [29]. It does not demonstrate that ferroptosis is upstream of OPN/MCP-1 induction, because direct temporal and pathway-specific tests were not performed. The results should therefore be interpreted as an *in vitro* association that warrants validation in animal models and human-relevant systems.

The functional role of PI3K/AKT signaling in CaOx-associated injury appears to be context dependent. Previous studies have reported cytoprotective PI3K/AKT responses in several CaOx/oxalate-related experimental settings, including studies of taurine, puerarin, and OATF [30–32], whereas injury-associated PI3K/AKT activation has also been observed in oxalate-related settings [33]. In the present NRK-52E model, CaOx increased the p-PI3K/PI3K and p-AKT/AKT ratios, whereas LY294002 attenuated several oxidative stress- and ferroptosis-related readouts. These pharmacological findings favor an injury-associated role of PI3K/AKT signaling under the present experimental conditions. However, the divergent findings in the literature suggest that the functional direction of this pathway may depend on cell type, injury context, treatment conditions, and experimental model. Our data therefore support pharmacological involvement of PI3K/AKT in this model but do not establish whether it lies upstream or downstream of NOX or whether the same functional direction applies *in vivo*.

This study has several limitations. First, all experiments were conducted in NRK-52E cells, and no animal, primary human tubular-cell, or clinical validation was performed. Accordingly, the present findings should be interpreted as *in vitro* pharmacological evidence and cannot be directly extrapolated to nephrolithiasis *in vivo* or in patients. Second, although CaOx increased NOX activity and NOX4 expression and GKT137831 attenuated several injury-associated readouts, we did not use isoform-specific genetic inhibition and cannot assign a sole causal role to NOX4. Third, Fer-1 and Erastin do not rule out apoptosis, necroptosis, or other death programs; comparative interventions and dedicated markers are needed. Fourth, the pharmacological studies do not establish a linear NOX–PI3K/AKT causal hierarchy. Finally, the transcriptomic findings were not followed by qPCR validation of selected individual differentially expressed genes, although ferroptosis- and PI3K/AKT-related pathway-level observations were further examined using protein-level and pharmacological approaches. These issues define the scope of the present conclusions.

Despite these limitations, the results support NOX-associated oxidative stress, ferroptosis-related injury, and PI3K/AKT pathway involvement as concurrent features of CaOx-associated injury in NRK-52E cells. Further validation in animal models, primary human renal tubular cells, and clinically relevant samples, together with genetic mechanistic studies, will be required before NOX- or PI3K/AKT-related pathways can be considered potential therapeutic targets for nephrolithiasis.

## 5. Conclusions

Calcium oxalate exposure in NRK-52E cells was associated with increased NOX activity and NOX4 mRNA and protein expression, ROS accumulation, reduced GSH and NADPH, increased Fe^2+^ and MDA, decreased GPX4, and increased ACSL4. Fer-1/Erastin and GKT137831/LY294002 experiments support pharmacological involvement of ferroptosis-related processes, NOX, and PI3K/AKT signaling. These *in vitro* data do not establish a complete causal pathway, exclude other regulated cell-death programs, or demonstrate relevance to animal or human nephrolithiasis.

## Data Availability

The datasets generated and/or analyzed during the current study, including the transcriptomic data, are available from the corresponding author upon reasonable request.
